# A case report of malignant struma ovarii with papillary thyroid carcinoma

**DOI:** 10.1002/ccr3.8610

**Published:** 2024-04-12

**Authors:** Yekta Rahimi, Soghra Rabizadeh, Sara Seifouri, Kiana Seifouri, Samane Salarvand, Manouchehr Nakhjavani

**Affiliations:** ^1^ University of Southern California Los Angeles California USA; ^2^ Endocrinology and Metabolism Research Center (EMRC), Vali‐Asr Hospital Tehran University of Medical Sciences Tehran Iran; ^3^ Guy's Hospital London UK; ^4^ Department of pathology Cancer Institute Tehran University of Medical Sciences Tehran Iran

**Keywords:** malignancy, papillary thyroid carcinoma, radioactive iodine ablation, Struma ovarii, total thyroidectomy

## Abstract

**Key clinical message:**

Struma ovarii (SO), is a rare and specialized ovarian teratoma. The treatment is controversial depending on the risk of recurrence and metastasis. Here a SO with papillary thyroid carcinoma is reported and the approach is thoroughly discussed.

**Abstract:**

Struma ovarii (SO) is a highly specialized ovarian teratoma primarily composed of thyroid tissue. Clinical features associated with SO include lower abdominal discomfort, unusual vaginal bleeding, ascites, and hyperthyroidism. While SO rarely transforms into malignancy, the optimal degree of treatment remains controversial due to the varying risks of recurrence and metastasis. In this report, we present the case of a 64‐year‐old woman experiencing abdominal pain and diagnosed with SO, accompanied by papillary thyroid carcinoma. We thoroughly discuss the evaluation and management of this rare condition.

## Graphical abstract

Graphical Abstract

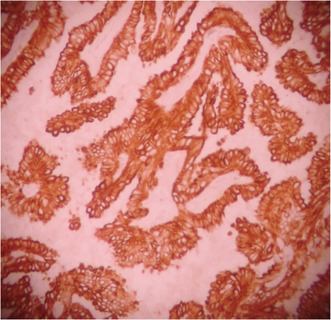

## 1 INTRODUCTION

Struma ovarii (SO) is a highly specialized ovarian teratoma, mainly composed of thyroid tissue.^1^, ^2^ It can be characterized by clinical features such as lower abdominal discomfort, unusual vaginal bleeding, ascites, and hyperthyroidism, and may be associated with an underlying thyroid disease.^3^, ^4^, ^5^, ^6^ Based on evidence patients with SO mostly have no symptoms or may present with non‐specific symptoms similar to other ovarian cancers.^7^, ^8^ It has been shown that 5% of SO cases are malignant, and metastases are not well‐documented. The transformation of struma into malignant thyroid tissue requires a BRAF genetic mutation, similar to the mutation that occurs in the thyroid, leading to malignancy.^9^ The papillary type is also the most common malignancy in SO.^10^ Nuclear features found in primary thyroid cancer, such as grooves, granular chromatin, and psammoma bodies, may also be observed in malignant ovarian masses.^11^ Approximately 70% of follicular cell‐derived thyroid carcinomas exhibit mutations in genes such as BRAF, RAS, RET, and NTRK1. Specifically, the BRAF mutation is observed in 29%–69% of primary papillary thyroid cancers.^4^ Despite these genetic associations, papillary thyroid carcinoma has an excellent surveillance record, mirroring its favorable prognosis. The 5 and 10‐year prognosis for malignant SO are 92% and 79%, respectively^12^ In the literature, no optimum treatment has been declared for malignant SO.^13^ Therefore, we present a rare case of SO and discuss its management.

## 2 CASE REPORT

### 2.1 Case history and physical examination

A 64‐year‐old woman presented with abdominal pain in 2014. She had experienced dull abdominal pain for some time, with no other specific symptoms. Physical examination revealed no palpable mass, and tenderness was negative. The patient had no significant medical or drug history.

### 2.2 Methods

Abdominopelvic ultrasonography revealed a large, heterogeneous mass with an irregular border (43 × 45 mm) in the left ovary. Hematological and biochemical investigations were normal: LDH (309 IU/mL, reference range <480), CEA (0.2 μg/mL, reference range 0–5.5), and Alpha‐fetoprotein (2.1 IU/mL, reference range <5).

Magnetic resonance imaging of the abdomen and pelvis suggested a large multiocular cystic lesion with a solid component in the left ovary, strongly indicating left ovarian cancer. Subsequently, a hysterectomy with bilateral salpingo‐oophorectomy was performed.

Pathological examination revealed a 1.5 cm endometrial polyp and a normal right ovary. The left ovarian mass was identified as a teratoma consisting of papillary thyroid carcinoma (Figure 2). The tumor comprised cartilage, bone, ciliated columnar epithelium, foci of normal thyroid follicles, and a papillary neoplasm with loose connective tissue and a vascular core lined with atypical cells demonstrating nuclear crowding, overlapping, and clearing.

**FIGURE 1:**
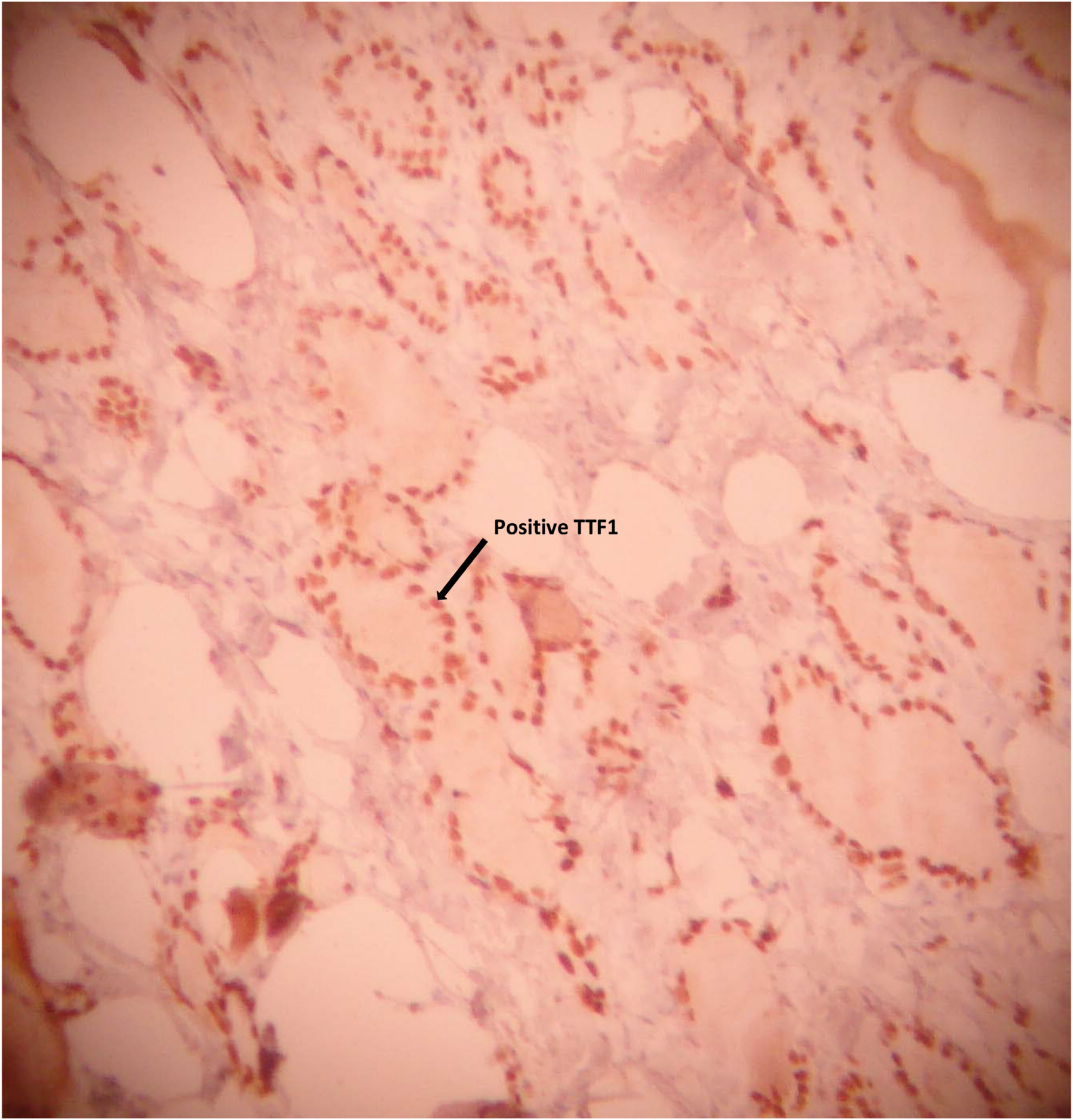
Immunohistochemical staining for TTF1 highlights thyroidal origin of neoplastic cells in struma ovarii (×100).

**FIGURE 2:**
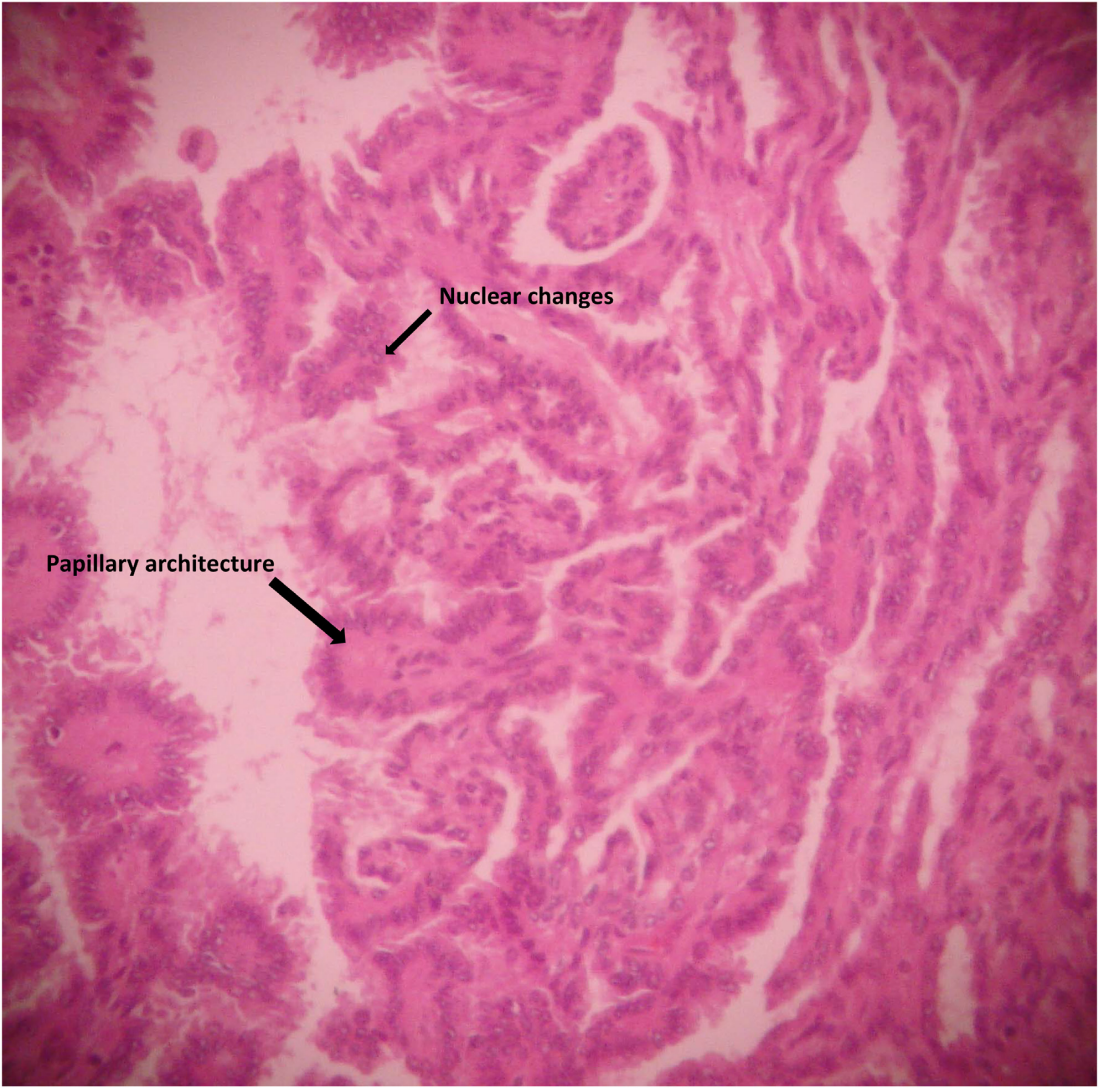
Focus of papillary thyroid carcinoma arising on struma ovarii. Note true papillae with multiple branching and nuclear changes of neoplastic cells including nuclear enlargement, crowding, overlapping, clearing, and inclusions (×100).

**FIGURE 3:**
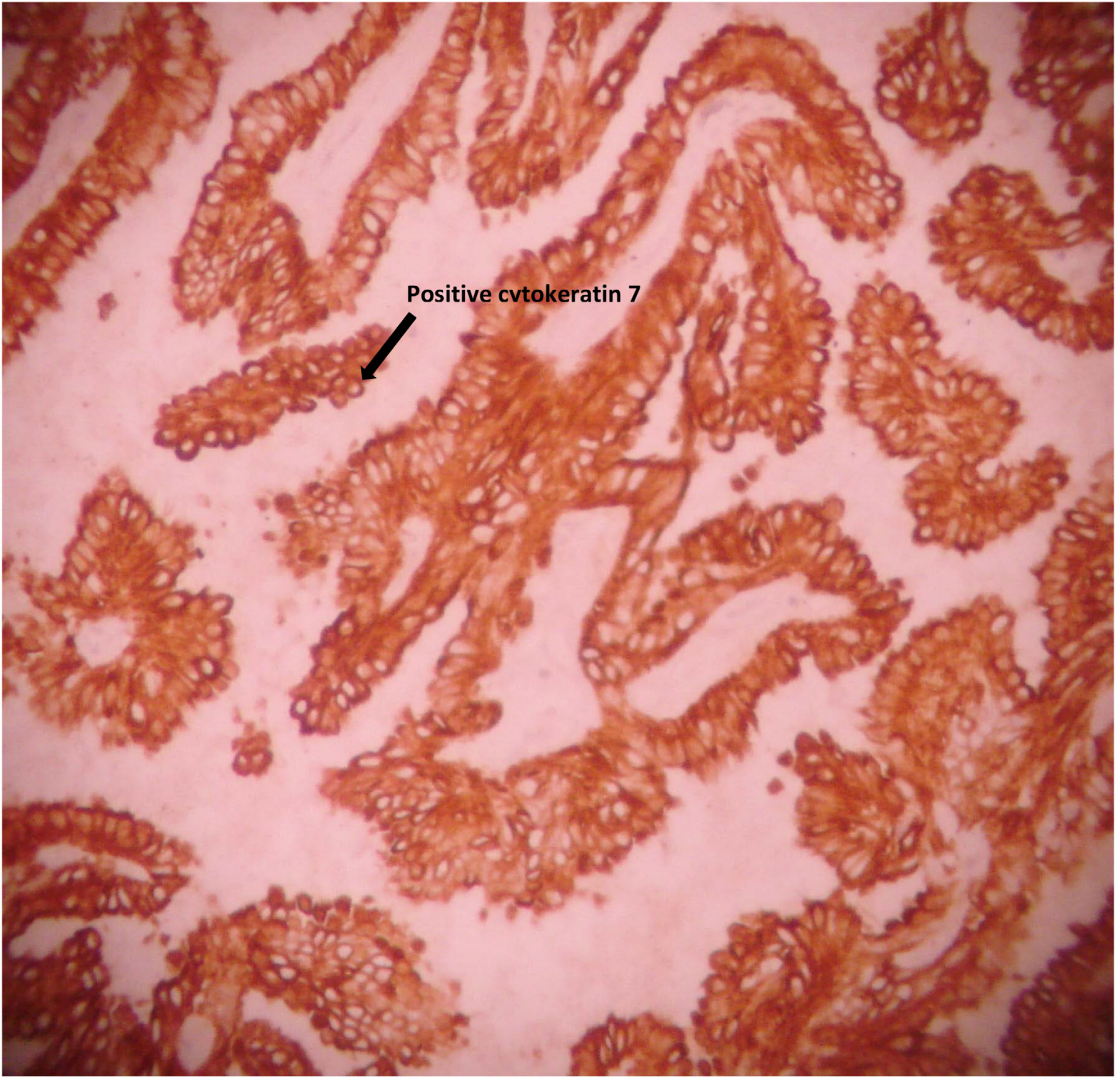
Immunohistochemical staining for cytokeratin seven shows a positive reaction in neoplastic cells in struma ovarii (×400).

Immunohistochemical (IHC) study yielded positive results for CK7, TTF1, and thyroglobulin, and negative for CA125. BRAF was positive in our patient. These IHC findings suggested that the ovarian teratoma was composed of thyroid tissue (Figures 1, 2, 3).

Laboratory results are summarized in Table 1: TSH: 4.4 mIU/L (0.5–5), T4:7.9 mcg/dL(4.7–12.5), T3: 1.3 ng/mL (0.8–2.3), thyroglobulin: 24.5 ng/mL, anti‐thyroglobulin:6.2 IU/mL. WBC: 4600 (4500–10,500), Hemoglobin: 14 g/dL (13.8–17.2), platelet: 337000/mL of blood (150,000–450,000), FBS: 115 mg/dL (<100), BUN: 21 mg/dL (6–24), Creatinine: 1.1 mg/dL (0.7–1.3), AST: 24 U/L (8–33), ALT: 50 U/L (7–55), Alk P: 335 U/L (44–147).

**TABLE 1:** Laboratory case results.

Laboratory tests	Conventional units	Reference range values	Patient results (2020)
LDH	IU/mL	<480	309
CEA	μg/mL	0–5.5	0.2
Alfa fetoprotein	IU/mL	<5	2.1
White blood cell	×1/μL	4500–10,500	4600
Count			
FBS	mg/dl	<100	115
Hemoglobin	g/dL	13.8–17.2	14
BUN	mg/dl	6–24	21
Platelet count	×1/μL	150,000–450,000	337,000
Creatinine	mg/dl	0.7–1.3	1.1
ALT	U/L	7–55	50
AST	U/L	8–33	24
ALK P	U/L	44–147	335
TSH	μIU/mL	0.5–5	4.4
T4	mcg/dl	4.7–12.5	7.9
T3	ng/mL	0.8–2.3	1.3

Abbreviations: ALK P, alkaline phosphatase; ALT, alanine transaminase; AST, aspartate aminotransferase; BUN, blood urea nitrogen; CEA, carcinoembryonic antigen; FBS, fasting blood sugar; LDH, lactate dehydrogenase; T3, triiodothyronine; T4, thyroxine; TSH, thyroid stimulating hormone.

The whole‐body scan conducted 3 h after the intravenous administration of 740 MBq of T99‐MIBI showed no evidence of abnormal MIBI‐avid lesions. Thyroid ultrasonography revealed an 8 mm nodule in the right lobe and a 28 × 16 mm lymph node in the right cervical region.

### 2.3 Conclusion and results

Following a total thyroidectomy, pathology results were negative for malignancy. Two months post‐thyroidectomy, laboratory tests indicated TSH >100 mIU/L, Tg: 2.05 ng/mL, and anti‐Tg: 3 IU/mL. Subsequently, the patient underwent ablation with 150 mCi of radioactive iodine (RAI). The post‐therapeutic I‐131 whole‐body scan revealed no uptake in the pelvic area or distant metastasis. The patient was prescribed a daily dose of levothyroxine at 150 μg.

Suppressive therapy. In the follow‐up, in 2017, laboratory test results were as follows: Tg <0.2 ng/mL, anti‐Tg <20 IU/mL, TSH:0.05 mIU/mL. Ultrasonography, of the neck showed three suspicious lymph nodes up to 14 × 8 mm on the left side. A biopsy of the lymph node was done and it was negative for malignancy. The patient had a good condition with no evidence of metastasis at other sites.

## 3 DISCUSSION

SO accounts for about 1% of all ovarian tumors and 2.7% of dermoid tumors^1^, ^2^ Malignant SO is exceptionally rare and lacks distinctive signs and symptoms. Patients with truma ovarii typically present with symptoms unrelated to the thyroid, such as abdominal mass, vague pain, or swelling; however, some may exhibit symptoms of hyperthyroidism.^14^ In a study conducted from 1940 to 2008, 60 out of 88 patients with malignant SO exhibited benign histological features, while 28 patients showed malignant histological features. Among the latter, 20 were diagnosed with papillary carcinoma. The majority of these individuals, typically between 40 and 50 years old, presented with chief complaints of abdominal pain, mass, or vomiting. In most cases, the mass was unilateral, with an average size of 13 cm. Similarly, we present the case of a 64‐year‐old woman who complained of abdominal pain, and upon examination, a tumor with a size of 4 × 4 cm was identified. The presence of thyroid follicles with colloid, resembling normal thyroid tissue, and the reaction to thyroglobulin antibodies and thyroid transcription factor‐1 in ovaries suggest normal SO. However, diagnosing malignant SO can be challenging. In Schmidt's study, four out of every six malignant struma cases exhibited BRAF mutation, while none of the nine benign struma cases were BRAF positive.^15^ Our patient's IHC results were positive for CK7, TTF1, and Thyroglobulin, and negative for CA125. As there are no specific guidelines for the treatment of malignant SO, decisions should be individualized based on the histological and pathological findings of the mass. According to Kabukcuoglu et al., pelvic surgery is deemed sufficient in these patients, and prophylactic thyroidectomy is considered unnecessary.^16^

In the studies conducted by Devaney et al. and Terayama et al., none of the patients with malignant SO received adjuvant therapy, and there was no clinical evidence of recurrence.^17^, ^18^ Some studies suggested that the RAI didn't improve mortality rate and overall survival rate.^19^, ^20^ However, it should be considered that the incidence of papillary carcinoma and malignant SO is increasing. To mitigate the chance of recurrence, it is recommended to perform thyroidectomy followed by RAI for tumors larger than 2 cm with distant metastasis, the presence of BRAF mutation, or synchronized thyroid cancer.^1^, ^9^, ^21^, ^22^, ^23^ Given the large size and BRAF‐positive tumor, despite the absence of synchronized thyroid cancer or metastasis, the following treatment was applied: total thyroidectomy, bilateral oophorectomy, and radioiodine ablation. In the follow‐up series, there were no specific signs of recurrence. In support of our management, no recurrence was observed in patients after total thyroidectomy and RAI ablation, even after 36 years.^24^ Other studies also recommend near‐total thyroidectomy followed by RAI ablation for the treatment of malignant SO^3^, ^9^, ^25^ Wu et al. presented a case with massive peritoneal metastasis 14 years after chemotherapy for malignant SO.^26^ The measurement of thyroglobulin and Tg antibodies, coupled with pelvic and thyroid imaging, can serve as biomarkers for detecting metastasis and relapse. Therefore, any detectable thyroglobulin in serum indicates the need for further assessment for recurrence.^27^, ^28^ In our case, thyroglobulin (Tg) and anti‐thyroglobulin (anti‐Tg) were measured, and the results were normal. There was no sign of recurrence after 3 years. The choice of an effective therapeutic approach may be influenced by factors such as the type of carcinoma, the age of the patient, and evidence of metastasis.^20^

In conclusion, we reported a rare case of malignant SO. The treatment procedure for malignant SO may vary depending on the stage of the disease. In the presented case, primary thyroid carcinoma treatment was applied, and no metastasis was detected after 3 years.

## AUTHOR CONTRIBUTIONS

**Yekta Rahimi:** Writing – original draft. **Soghra Rabizadeh:** Supervision; validation. **Sara Seifouri:** Writing – review and editing. **Kiana Seifouri:** Writing – review and editing. **Samane Salarvand:** Visualization. **Manouchehr Nakhjavani:** Conceptualization; project administration; supervision; validation.

## FUNDING INFORMATION

None.

## CONFLICT OF INTEREST STATEMENT

The authors confirm that there is no conflict of interest.

## ETHICS STATEMENT

Written informed consent was obtained from the patient to publish this report in accordance with the journal's patient consent policy.

## CONSENT

Written informed consent was obtained from the patient to publish this report in accordance with the journal's patient consent policy.

## Data Availability

The data that support the findings of this study are available on request from the corresponding author.
